# Effects of reduced salinity caused by reclamation on population and physiological characteristics of the sesarmid crab *Chiromantes dehaani*

**DOI:** 10.1038/s41598-022-05639-4

**Published:** 2022-01-31

**Authors:** Weiwei Lv, Quan Yuan, Weiwei Huang, Xiaolin Sun, Wenzong Zhou, Yunlong Zhao

**Affiliations:** 1 Eco-Environmental Protection Research Institute, Shanghai Academy of Agricultural Sciences, Shanghai, 201403 China; 2 Shanghai Runzhuang Agricultural Technology Co., Ltd, Shanghai, 201403 China; 3 School of Life Sciences, East China Normal University, Shanghai, 200241 China

**Keywords:** Population dynamics, Wetlands ecology

## Abstract

Reduced salinity is a major factor that causes macrobenthic degradation in reclaimed wetlands. We investigated populations of the sesarmid crab *Chiromantes dehaani* in reclaimed and natural wetlands. Then, in the laboratory, we exposed male and female crabs to four salinity levels (0, 6, 12 and 18) for 96 h to analyse the effects of reduced salinity on osmoregulatory enzyme activities in the posterior gills and digestive and immune enzyme activities in the hepatopancreas of *C. dehaani*. The results revealed a significant positive correlation between the number of crabs and salinity. In the laboratory, we found that the isosmotic point of *C. dehaani* was close to 16 ppt. The crabs showed strong hyper-osmotic regulation when exposed to 0–6 ppt salinities. Moreover, in this salinity range, amylase activities were significantly inhibited. Under low-salinity stress, the immune enzyme activities were significantly activated. However, phenoloxidase and lysozyme activities were inhibited in the freshwater environment. The male and female crabs showed no significant differences in most of the enzyme activities. Thus, reduced salinity can adversely affect the digestive and immune functions of *C. dehaani*, which may cause population degradation in reclaimed wetlands. Our findings can provide new insights into the effects of reclamation on macrobenthos.

## Introduction

Shanghai is one of the most prosperous cities in China; however, the development of this city has been restricted by land scarcity. Wetland reclamation in the Yangtze Estuary has been considered as one of the main solutions to land scarcity. From the 1960s to 2015, the area of reclaimed land in the Yangtze Estuary has increased by 543.9 km^2^^1^. A major risk associated with reclamation is the loss of macrobenthic diversity. Previous studies have shown that reclamation projects can cause water salinity changes in intertidal wetlands^2^. Thus, many scholars have speculated that the reduced salinity caused by reclamation may be one of the main reasons for the degradation of macrobenthic communities^3,4^.

The sesarmid crab *Chiromantes dehaani* is a dominant crab in the mudflats of the Yangtze Estuary^5^. This crab plays ecologically important roles as a secondary consumer in intertidal ecosystems^6^. *C. dehaani* prefers to live in brackish water to meet its salinity requirements for reproduction and larval development^7^. However, like other macrobenthos, *C. dehaani* populations have been seriously disturbed by reclamation^8^. Currently, there is limited information on why *C. dehaani* cannot survive in low-salinity reclaimed areas because few studies have investigated the crab physiological traits altered by salinity changes in reclaimed wetlands.

Usually, estuarine species can regulate osmotic pressure, which can contribute to maintaining the osmotic concentration of their body fluids during salinity challenges^9–11^. Many studies have shown that euryhaline crabs mainly rely on the posterior gills to transport ions and maintain haemolymph osmotic pressure and membrane potential balance^12–15^. In the gill epithelium, Na^+^ and Cl^−^ enter ionocytes via Na^+^/H^+^ and Cl^−^/HCO_3_^−^ exchangers, respectively, at the apical membrane of ionocytes. In this process, the activity of Na^+^/K^+^-ATPase plays an important role for NaCl absorption via the gills^16^. Thus, the occurrence of osmoregulationis based on efficient ionic regulation (mainly of Na^+^ and CI^−^) and increased levels of Na^+^/K^+^-ATPase activity^17^.

In euryhaline aquatic animals, osmoregulation during salinity acclimation may cause changes in digestive enzyme activities^18^. A possible explanation is that aquatic animals undergo metabolic reorganization to meet the increasing energy requirements due to salinity changes^19^. Digestive enzymes play an important role in the metabolic regulation mechanism of organisms. Because many crustaceans are omnivorous, the digestive enzymes associated with carbohydrates (e.g. amylase and cellulase), lipids (e.g. lipase) and proteins (e.g. pepsin and trypsin) have been systematically studied^19,20^. Previous studies have demonstrated that salinity acclimation can promote digestive enzyme activities in euryhaline decapods. Moreover, the effect of salinity change on digestive enzyme activity is usually related to the intensity and mode of acclimation^21^.

Salinity acclimation may also affect the immune systems of aquatic animals. Previous studies have demonstrated that the activity of immune enzymes such as phenoloxidase, alkaline phosphatase and lysozyme in crustaceans is modified in response to salinity stress. The nonspecific immune system of crustaceans includes cellular and humoral immunity. Phenoloxidase is associated with the phenoloxidase activating system, which plays an important role in humoral immunity. When this system is activated, the released phenoloxidase can inhibit and kill pathogens to achieve the effect of immunity^22^. Phosphatase is an important part of the lysosomal enzyme in macrophages, which can cleave and eliminate foreign bodies by forming the hydrolase system^23^. Lysozyme in bactericidal phagocytes breaks down the cell wall of gram-positive bacteria by hydrolysis of the β-1,4-glycosidic bond^24^.

In this study, we investigated the number of *C. dehaani* in different habitats in reclaimed wetlands in the Yangtze Estuary. We simulated the changes in water salinity in reclaimed wetlands and measured the activities of osmoregulatory, digestive and immune enzymes. The primary objective of this study was to detect the effects of reduced salinity caused by reclamation on the population and physiological characteristics of *C. dehaani*.

## Results

### Water salinity and population characteristics

**Figure 1:**
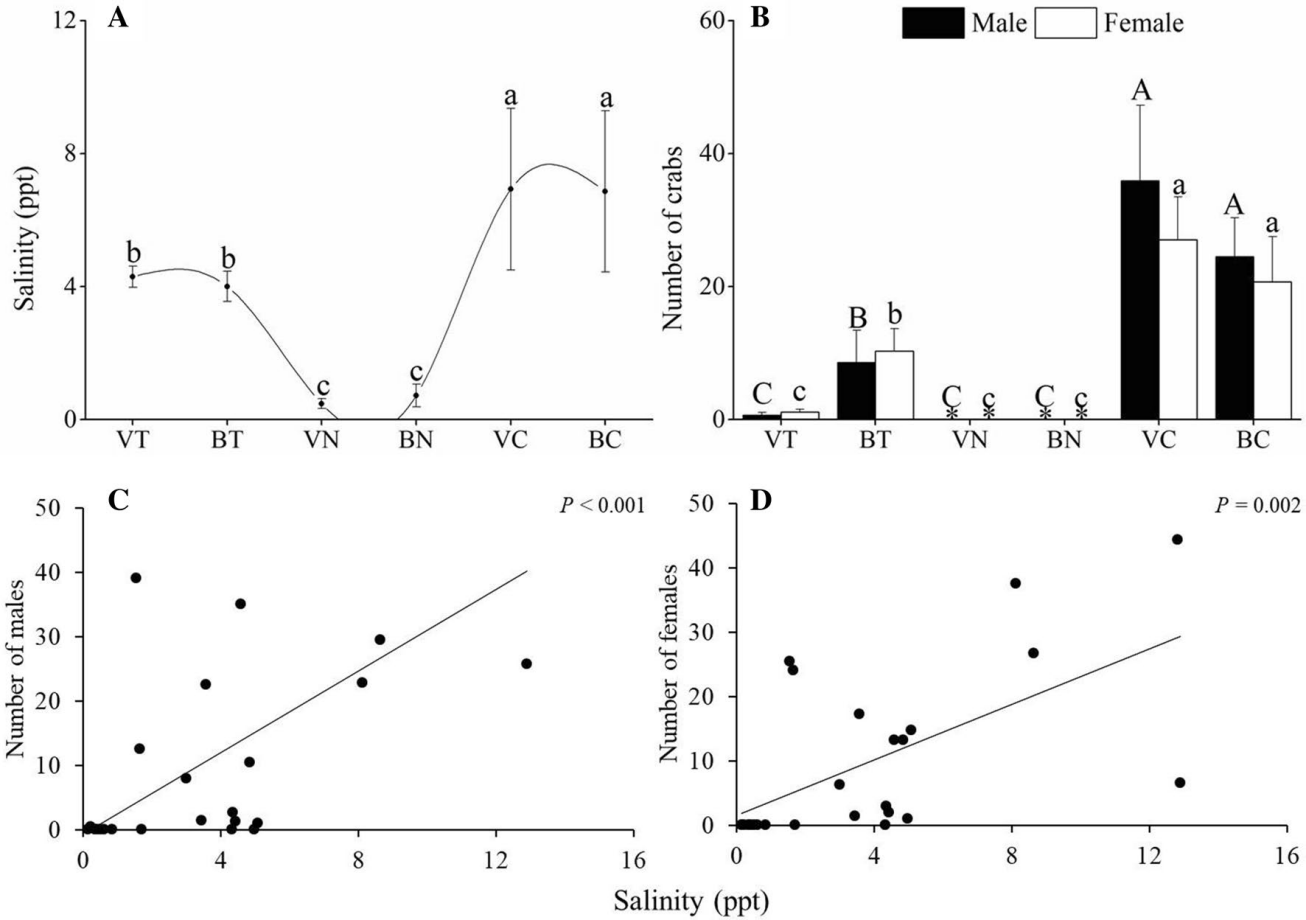
Water salinity **(A)** and number of *C. dehaani* crabs **(B)** in six sampling areas. Values are mean ± SE; N = 4. The correlations between water salinity and numbers of male **(C)** and female **(D)** crabs. The abbreviations represent sampling sites: VT and BT, vegetated and bald sites in tidal-affected region; VN and BN, vegetated and bald sites in non–tidal-affected region; VC and BC, vegetated and bald sites BC in natural tidal flat. ^*^ indicates that the number of crabs was 0.

The changes in water salinity and numbers of female and male crabs are shown in Fig. 1A,B. The water salinities were significantly higher at VT (3.5–5.0 ppt) and BT (3.0–5.1 ppt) sites than at VN (0.1–0.9 ppt) and BN (0.2–1.7 ppt) sites (*P* < 0.05). However, the water salinities at VT and BT sites were significantly lower than those at VC (1.7–12.8 ppt) and BC (1.5–12.9 ppt) sites (*P* < 0.05).

The numbers of male and female crabs were significantly higher at the BT site (8.7 ± 0.3, 10.3 ± 3.4 individuals) than other sites in the reclaimed zone (*P* < 0.05). In contrast, the BT site had significantly lower numbers of male and female crabs than VC (35.9 ± 11.3, 27.1 ± 6.4 individuals) and BC (24.5 ± 5.9, 20.7 ± 6.8 individuals) sites (*P* < 0.05). No significant differences were observed in the numbers of male and female crabs at all sites (*P* > 0.05), and no crabs were found at the VN and BN sites.

The correlation between water salinity and numbers of female (regression analysis, y = 3.1619x − 0.6417, r = 0.669, *P* < 0.001, Fig. 1C) and male (regression analysis, y = 2.1535x + 1.5028, r = 0.601, *P* = 0.002, Fig. 1D) crabs was significant.

### Haemolymph osmolality and Na^+^/K^+^ ATPase activity in the posterior gills

**Figure 2:**
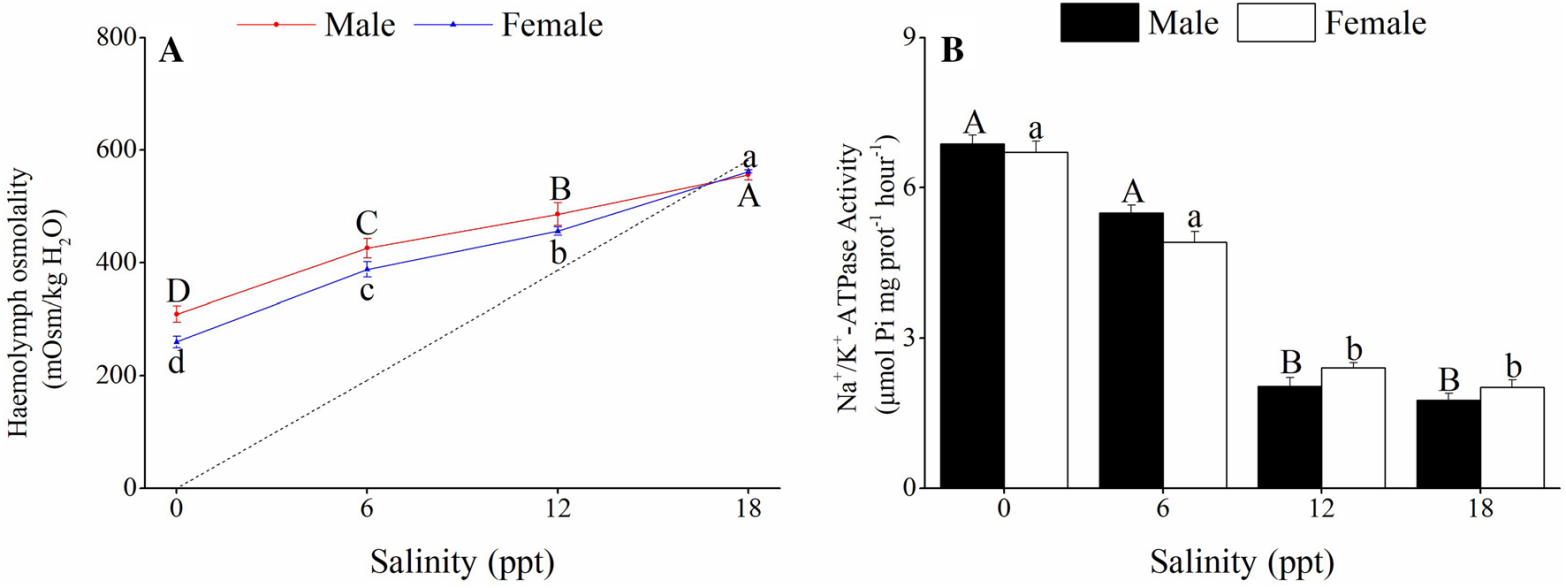
Haemolymph osmolality **(A)** and gill Na^+^/K^+^ ATPase activities **(B)** of *C. dehaani* crabs after exposure to salinity changes for 96 h. Values are mean ± SE, N = 4. Different letters above the data indicate significant differences between the salinity treatments, as assessed using Tukey’s tests (*P* < 0.05).

No mortality was observed in all treatments during the experiment period. There were significant differences in haemolymph osmolality and Na^+^/K^+^ ATPase activities in the posterior gills between the female and male crabs (*P* > 0.05; Fig. 2A). After 96 h of exposure, haemolymph osmolality of the crabs was significantly higher in the groups exposed to 12 and 18 ppt salinity than in those exposed to 0 and 6 ppt salinity (*P* < 0.05). The crabs were hyper-osmoregulators at salinities ranging from 0 to 12 ppt, but the crabs acted as hypo-osmoregulators at 18 ppt. The isosmotic point was calculated at ∼16 ppt salinity.

The activities of Na^+^/K^+^ ATPase in the posterior gills of the female and male crabs were significantly higher at 0–6 ppt salinities than at 12–18 ppt salinities (*P* < 0.05; Fig. 2B). However, no significant difference in Na^+^/K^+^ ATPase activity was detected when the crabs were exposed to 0–6 ppt and 12–18 ppt salinities (*P* < 0.05).

### Digestive enzyme activities in the hepatopancreas

**Figure 3:**
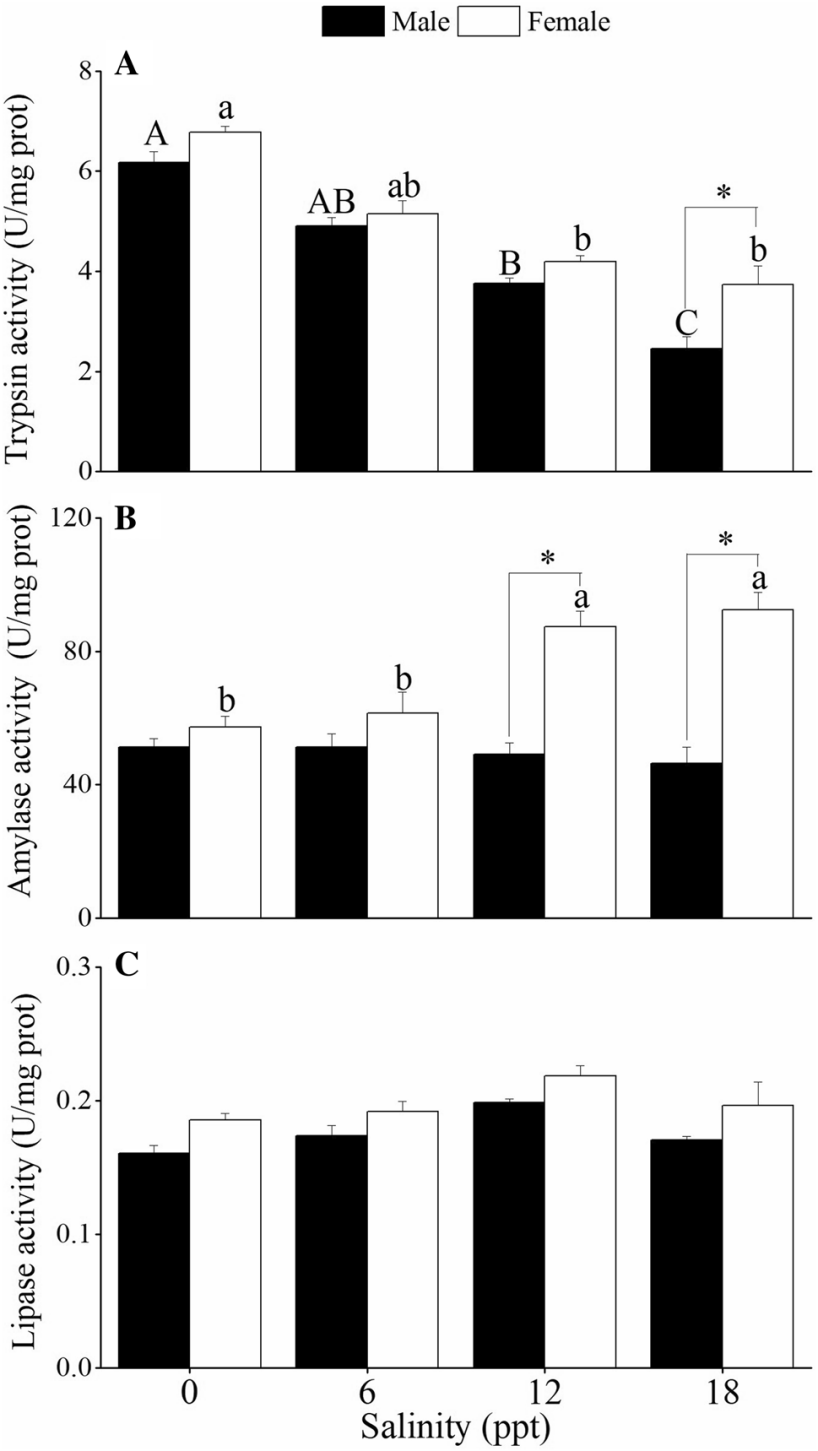
Effects of salinity exposure on the activities of trypsin **(A)**, amylase **(B)** and lipase **(C)** in the hepatopancreas of male and female *C. dehaani* crabs. Values are mean ± SE, N = 4. Different letters above the data indicate significant differences between the salinity treatments, as assessed using Tukey’s tests (*P* < 0.05). * indicates significant differences between female and male crabs, as assessed using Tukey’s tests (*P* < 0.05).

Figure 3 shows digestive enzyme activities in the hepatopancreas of *C. dehaani* after the salinity treatments for 96 h. Trypsin activity in both female and male crabs showed a significant decrease as the salinity level increased (*P* < 0.05; Fig. 3A). Moreover, trypsin activity of the female crabs was significantly higher than that of the males at 18 ppt salinity (*P* < 0.05). Amylase activities of the female crabs were significantly higher at 12–18 ppt than at 0–6 ppt salinities (*P* < 0.05; Fig. 3B). However, no significant differences were detected in the amylase activities of male crabs in the four salinity treatment groups (*P* > 0.05). The female crabs exposed to 12–18 ppt salinities showed significantly higher amylase activities than the males (*P* < 0.05). Lipase activities of the crabs were not significantly different with respect to sex and salinity treatments (*P* > 0.05; Fig. 3C).

### Immune enzyme activities in the hepatopancreas

**Figure 4:**
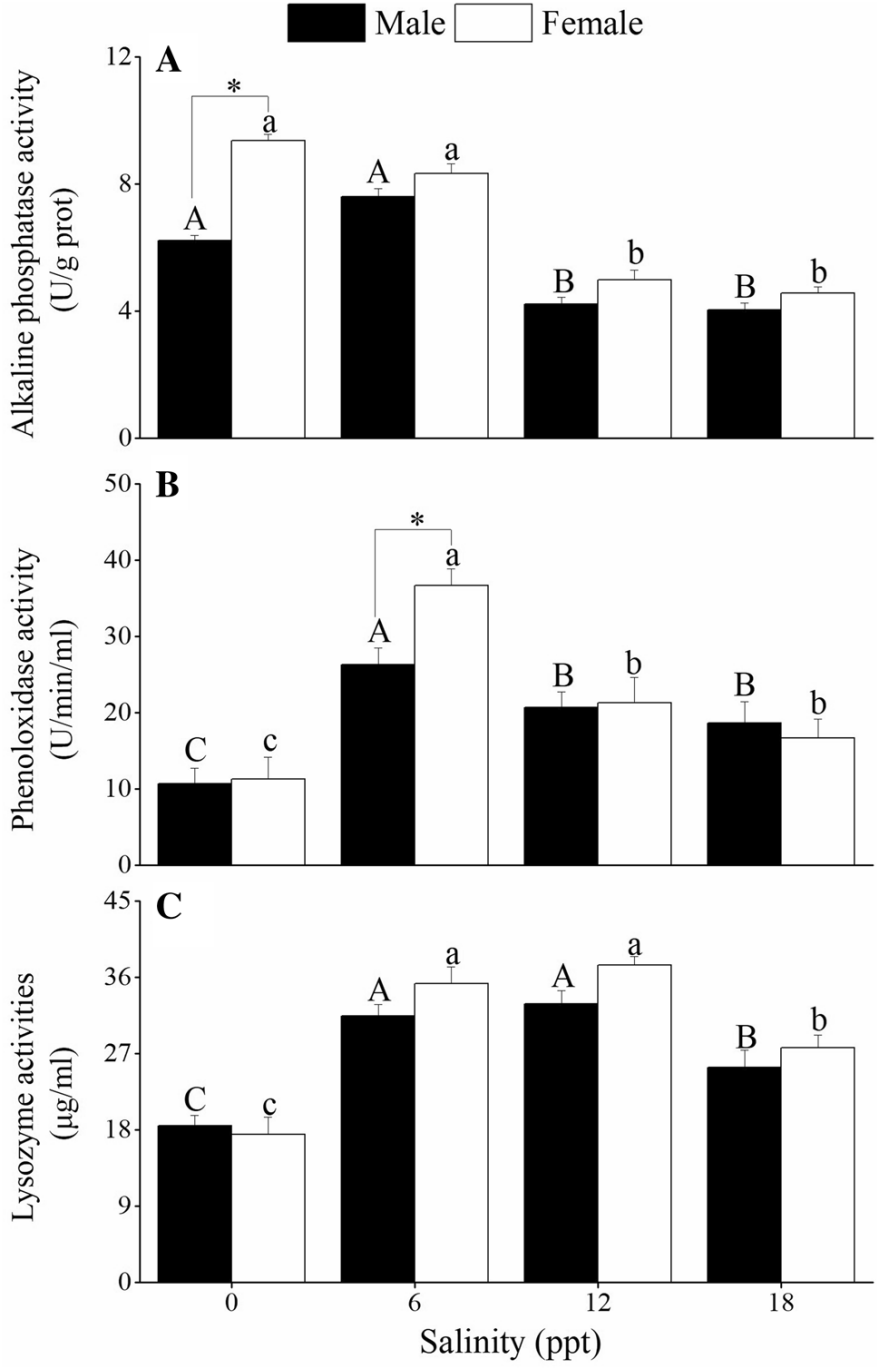
Effects of salinity exposure on the activities of alkaline phosphatase **(A)**, phenoloxidase **(B)** and lysozyme **(C)** in the hepatopancreas of male and female *C. dehaani* crabs. Values are mean ± SE, N = 4. Different letters above the data indicate significant differences between the salinity treatments, as assessed using Tukey’s tests (*P* < 0.05). * indicates significant differences between female and male crabs, as assessed using Tukey’s tests (*P* < 0.05).

Immune enzyme activities in the hepatopancreas of *C. dehaani* after 96 h of exposure are shown in Fig. 4. Alkaline phosphatase activities in the female and male crabs were significantly higher at 0–6 ppt salinities than at 12–18 ppt salinities (*P* < 0.05; Fig. 4A). Moreover, the female crabs showed significantly higher alkaline phosphatase activity than the male crabs at 0 ppt salinity (*P* < 0.05). Phenoloxidase activities in the male and female crabs decreased significantly from 6 to 12 ppt salinities after a significant increase at 0–6 ppt salinities (*P* < 0.05; Fig. 4B). The female crabs exposed to 6 ppt salinity showed significant higher phenoloxidase activity than the male crabs (*P* < 0.05). Lysozyme activities in the female and male crabs were significantly higher at 18 ppt salinity than at 0 ppt salinity, but significantly lower at 6–12 ppt salinities (*P* < 0.05; Fig. 4C). No significant differences in lysozyme activities were observed between the male and female crabs (*P* > 0.05).

## Discussion

In this study, *C. dehaani* crabs could not be collected from the non-tidal-affected zone, where the water salinity was extremely low (0.1–1.7 ppt). In contrast, in the nearby tidal-affected area, *C. dehaani* crabs were found at 3–5.1 ppt water salinity. The correlation analysis showed a significant positive correlation between the number of female and male crabs and water salinity. Therefore, water salinity is the key factor that affects the sustainable development of *C. dehaani* population. However, the number of crabs in the tidal-affected area was significantly lower than that in the natural tidal flat. This may be because of the adverse effects of reclamation on tidal salinity, which may cause long-term osmotic stress in *C. dehaani*.

The simulated experiments showed that the haemolymph osmolality of *C. dehaani* increased significantly as the salinity increased. Moreover, the osmoregulatory pattern of the crabs changed from hyper-osmoregulation to hypo-osmoregulation at salinities ranging from 12 to 18 ppt. These results indicate that *C. dehaani* has a strong ability to regulate osmolality during short periods of salinity exposure (96 h). In addition, the isosmotic point of *C. dehaani* was close to 16 ppt. This salinity level is higher than that observed in the natural tidal flat (1.5–13.0 ppt). Crustaceans may increase the isosmotic point of the haemolymph during gonadal development, which may contribute to the absorption of ions from the surrounding environment^25^. In this study, the crabs were collected in October, when they were in the gonadal development stage.

The activities of Na^+^/K^+^-ATPase are crucial for osmoregulation in decapod crustaceans. In this study, Na^+^/K^+^-ATPase activities in the posterior gills of *C. dehaani* were significantly activated by 0–6 ppt salinity. This suggests that *C. dehaani* crabs can actively reabsorb Na^+^ and Cl^-^ to replace the lost NaCl and maintain osmotic balance when entering a low-salinity or freshwater environment. In addition, in this study, no significant differences in Na^+^/K^+^-ATPase activity were found between the posterior gills of the male and female crabs, which is similar to the results of the freshwater crab *Eriocheir sinensis*^26^. Similar to our results, another study showed that female and male of the euryhaline crab *Neohelice* (*Chasmagnathus*) *granulata* have strong hypo-osmoregulatory abilities^27^. However, the differences in the osmoregulatory ability of euryhaline crabs due to sex may be affected by season and development stage^27,28^. In the Yangtze Estuary, the biomass of *C. dehaani* was usually the highest in autumn. During this season, fewer environmental conditions were not conducive to the survival of this crab. Thus, both female and male crabs may have enough energy to regulate osmotic pressure.

This study revealed that trypsin activities in the hepatopancreas of both male and female crabs were activated in the freshwater environment. This may be a physiological adjustment of *C. dehaani* to cope with low salt conditions. In crustaceans, proteins can be hydrolysed by proteases to form free amino acids, which may also contribute to the regulation of osmotic balance^28^. Trypsin is a proteolytic enzyme that cleaves peptide bonds in the carboxylic groups of arginine and lysine^29^. Previous studies have confirmed that arginine and lysine contents increased in aquatic animals under hyper-osmotic or hypo-osmotic stress^30,31^. However, there is a lack of definitive evidence to demonstrate the general correlation between trypsin and osmoregulation, so further studies are required to analyse whether the increasing trypsin activity could reflect a higher amino acid metabolism in attempt of the crab to obtain osmotic effectors under low salinity conditions.

The digestive enzyme activity was generally consistent with the feeding habitats^32^. In this study, we found relatively high amylase activities in the hepatopancreas of *C. dehaani* after 96 h of exposure. This result may seem contradictory, as mussel meat was the only food in the experiment. However, *C. dehaani* is a typical herbivorous macrobenthos^33^. In the tidal flat of the Yangtze Estuary, this crab mainly lives in the growing areas of *Phragmites australis* and *Spartina alterniflora* and feeds on the leaves of these plants. Thus, the high activity of amylase enzyme could be rather attributed to the previous secretion in the tidal flats where they fed mainly on hydrophytes. However, we also found extremely low lipase activity, which may be due to the low fat content of the diets or the substrate used in the experiment^19^. In addition, the digestive enzyme activities of the female crabs were generally higher than those of the male crabs, which is similar to the results of Ye^34^. The crabs were collected in October, which is a typical mating season^35^. This shows the possibility of deriving extra food energy to compensate for the energy loss of reproduction.

This study also revealed that the immune enzymes (i.e. alkaline phosphatase, phenoloxidase and lysozyme) in the male and female crabs were activated at 6 ppt salinity, which suggests that reduced salinity may trigger a temporary immune response in *C. dehaani*. Similar results have been found in *Penaeus japonicus*^36^, *Penaeus chinensis*^37^ and *Penaeus vannamei*^37^. In contrast, phenoloxidase and lysozyme activities were inhibited in the crabs exposed to 0 ppt salinity. Decapods usually exhibit an immune stress response from they move from an isotonic to non-isotonic environment, and it mainly manifests as an emergency response, metabolic adaptation or changes to the body’s immune capacity^38–40^. According to the intensity and time of exogenous stimulus and immune regulation characteristics of the animal body, stress response can be manifested as stress adaptation and stress injury. In this study, phenoloxidase and lysozyme activities of *C. dehaani* were inhibited in the freshwater environment, which indicates that the crabs may have been maladaptive and their immune resistance will continue to decline in the non–tidal-affected area of the reclaimed wetland.

Alkaline phosphatase, phenoloxidase and lysozyme activities in the hepatopancreas were higher in the female crabs than in the male crabs. Differences in the immune defence ability of decapods due to sex may be related to gonadal development and reproductive behaviour^41^. Female crabs may need to maintain a higher immune defence than male crabs to cope with the threat of constant changes in environmental salinity during reproduction. Gonadal development of the female crabs was relatively delayed when compared with that of the male crabs. The hepatopancreas is the main energy storage tissue of *C. dehaani*, and energy transfer during gonadal development may influence the activities of immune-related enzymes in the different sexes of *C. dehaani*.

On the basis of these results, *C. dehaani* cannot be well adapted to the reduced salinity in a reclaimed area, which may cause a decline in its population. In fact, a vast majority of the native macrobenthos will gradually disappear after the reclamation of tidal flats^8^. According to previous studies, the number of macrobenthos has decreased from 63 species in 1998–2000 to 40 species in 2011–2013 in the east shoal of Chongming Island^42^. The south bank of the Yangtze Estuary, another reclaimed wetland, lost 33 species of macrobenthos during the same period^42^. The Yangtze Estuary offers an overwintering habitat for thousands of migratory birds between East Asia and Australia^43^. The loss of macrobenthos, which are major secondary producers, is bound to have a negative impact on overwintering birds. Moreover, the reclamation may cause the loss of spawning and reproduction grounds for many commercial and protected species, such as *E. sinensis*^44^ and *Acipenser sinensis*^45^. The decrease in macrobenthic diversity could reduce the efficiency of material cycling and energy flow in the reclaimed wetlands. From the perspective of ecological security, the intensity of wetland reclamation in the Yangtze Estuary should be reduced appropriately. Natural tides can be introduced to regulate water salinity in reclaimed areas and promote the self-restoration of macrobenthic diversity.

## Conclusions

In this comprehensive study, we used both field and laboratory data to evaluate the effects of reduced salinity caused by reclamation on the population and physiological characteristics of *C. dehaani*. The results of the field investigation revealed that the *C. dehaani* population decline in reclaimed areas is closely related to decreased salinity. In the laboratory, the isosmotic points of female and male *C. dehaani* were detected at ∼16 ppt salinity. The crabs acted as hyper-osmoregulators in low-salinity and freshwater environments (0–6 ppt). Their ability to digest carbohydrates was impaired, but their ability to digest proteins was enhanced. Under low-salinity stress, the immune indices of *C. dehaani* were activated within a short period. However, some immune enzymes of the crabs were inhibited when they were exposed to the freshwater environment. The activities of digestive and immune enzymes were generally higher in the female crabs than in the male crabs. Thus, reduced salinity may adversely affect the digestive and immune processes of *C. dehaani*, which could explain the effects of reduced salinity on essential crab species in reclaimed wetlands.

## Materials and methods

### Field investigation

In this study, we investigated the number of *C. dehaani* and water salinity in a reclaimed area in the east shoal of Chongming Island. This wetland was enclosed in 2013, and several sluices were constructed in the seawalls during the project. The main functions of these seawalls were to regulate water level and salinity.

**Figure 5:**
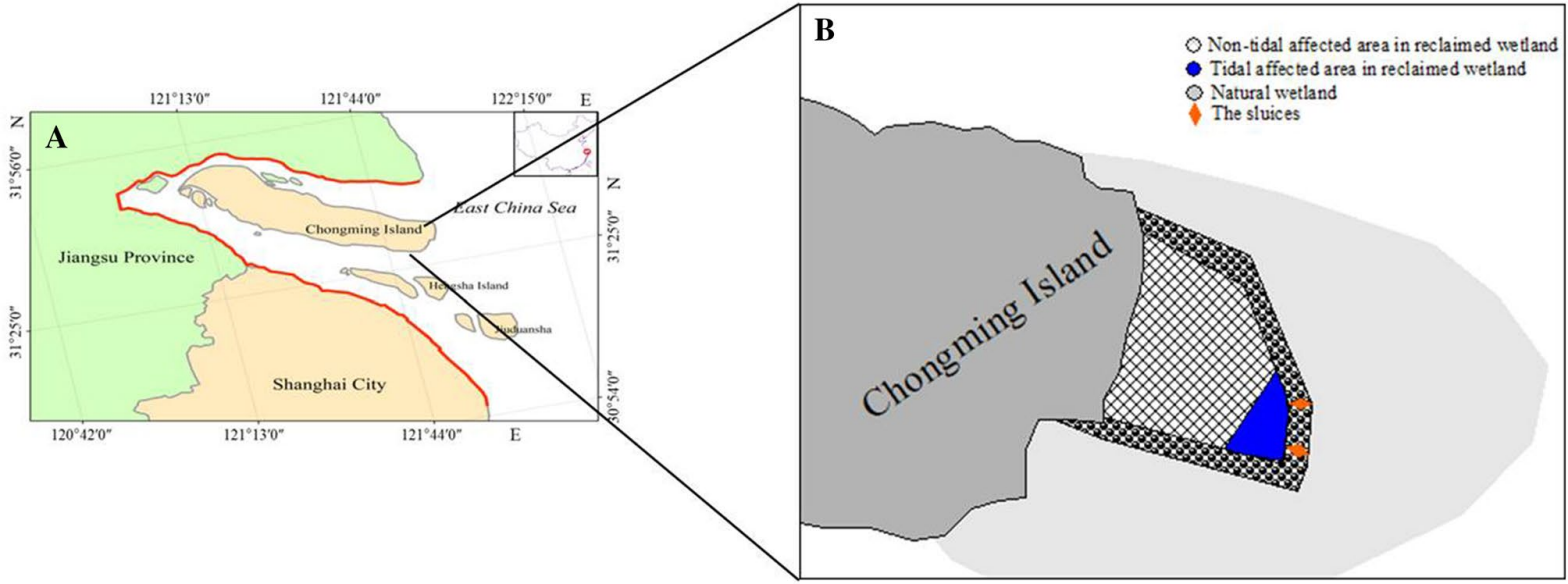
Location of the study area in the Yangtze Estuary in China **(A)** and the sampling areas in the east shoal of Chongming Island **(B)**.

*C. dehaani* crabs were collected from reclaimed and natural wetlands once a month in August and November in 2020 and January and March in 2021. A total of four sampling sites were distributed across the two survey regions: vegetated site (VT) and bald site (BT) in a tidal-affected region and vegetated site (VN) and bald site (BN) in a non–tidal-affected region. Two control sites were selected in a natural tidal flat: vegetated site (VC) and bald site (BC) (Fig. 5A,B). The catch-per-unit-effort (CPUE) method was used to collect *C. dehaani*. CPUE is defined as crab collection for 30 min by a person. In each sampling area, CPUE was repeated four times. The water salinity at each sampling area was measured using a Multi 350i water analyzer (WTW, MUC, Germany). The samples were transferred to the laboratory, where the crabs were counted and their sex was identified.

### Physiological experiment

*C. dehaani* adults in the intermoult stage (about 15–25 g) were purchased from Zhengyu farmers’ markets in October 2020. The experimental crabs were acclimated at Zhuanghang Experiment Station in Shanghai Academy of Agriculture Science (Shanghai, China) for one week. During the acclimation, all crabs were reared in cement pools with aerated artificial seawater at 12.0 ± 0.1 ppt salinity. Artificial seawater was prepared using marine salt dissolved in dechlorinated tap water. Water salinity was measured with the Multi 350i water analyzer. Throughout the acclimation period, all crabs were fed with freshwater mussels once a day. The water temperature and pH were maintained at 24 ± 1 ℃ and 8.2 ± 0.2, respectively.

After the acclimation period, female and male crabs were separately exposed to 0, 6, 12 and 18 ppt salinity treatments (four replications per treatment) in a stepwise manner (6 ppt every 3 h) to prevent physiological shock due to sudden changes. Each treatment group of 32 crabs (16 females and 16 males) was randomly placed in eight plastic containers, i.e. four crabs in each container. After 96 h, one crab from each container were anesthetized in the ice bath for 15 min. The haemolymph samples were extracted from the arthropodial membrane at the 3rd swimming legs by using a disposable sterile syringe and stored in a 2 mL centrifuge tube. Then, the hepatopancreas and posterior gills (6th and 7th gills) were removed and frozen in liquid nitrogen, and all samples were maintained at − 80 °C for further analysis.

The haemolymph osmolality was measured with a BS-88 freezing point osmometer (Ashtec, USA) and expressed in mOsm/kg H_2_O. The activities of Na^+^/K^+^ ATPase (One activity unit was defined as the amount of ATP decomposed by ATPase in tissue to produce 1 μmol inorganic phosphorus per milligram of tissue per hour at 37℃) in the posterior gills and lysozyme (One activity unit was defined as the increase in absorbance by the decomposition of bacterial solution per mL sample per two minutes at 37℃ and 530 nm), alkaline phosphatase (One activity unit was defined as 1 mg tissue protein that interacts with the matrix at 37℃ for 15 min and produces 1 mg of phenol) and lipase (At 37℃, each gram of tissue protein reacted with the substrate for 1 min, and each micromole of substrate consumed was defined as one enzyme activity unit) in the hepatopancreas were detected with commercial assay kits (Jiancheng Bioengineering Institute, Nanjing, China), according to the manufacturer’s instructions^46,47^. Before the test, 0.1 g gill or hepatopancreas sample was mixed with 900 mL normal saline (0.9%). Then, the mixture was thoroughly homogenized and centrifuged at 3500 rpm for 10 min (4 ℃). The supernatant was collected for further analyses.

Phenoloxidase activity was determined using the method of Ashida (1971). l-DOPA (Jiebes Biological Co., Ltd., Shanghai, China) was prepared with 0.01 mol/L l-DOPA solution (l-DOPA:HCL-K_3_PO_4_ = 0.002:1). HCl-K_3_PO_4_ was prepared by mixing 50 mL NaH_2_PO_4_-2H_2_O (0.2 mol/L) and 22 mL HCl (0.1 mol/L) in a 200 mL volumetric flask for constant volume. Then, 10 μL of the tissue homogenate to be tested and 10 μL of l-DOPA solution were added successively to the aseptic enzyme plate in a constant temperature water bath (28℃) for 40 min. Next, 200 μL of pre-cooled potassium phosphate buffer solution (pH  6.0) was added, and light absorption was measured. One phenoloxidase activity unit (U) was defined as 0.001 increase in OD_490_ per minute.

Trypsin activity was determined as follows: 100 μL of the tissue homogenate to be tested was put in a 2 mL centrifuge tube, and 500 μL of 0.5% casein solution, 25 μL of 0.04 mol/L EDTA-Na_2_, 100 μL of 0.05 mol/L borax-sodium hydroxide buffer solution (pH  9.8) and 150 μL of double distilled water were added to obtain a total volume of 875 μL. The solution was mixed in a water bath at 37 ℃ for 15 min. Then, 250 μL trichloroacetic acid (30%) was added, and the mixture was centrifuged at 1400 rpm for 10 min (4 ℃). The supernatant was used to determine tyrosine content with the l-phenol method. The tyrosine content was determined using colorimetry at 660 nm. Enzyme activity (U/mg prot) was defined as the catalytic hydrolysis of casein to 1 μg tyrosine by trypsin per minute per mg protein at 37 ℃.

Amylase activity was determined as follows: 100 mg starch was added to 10 mL phosphate buffer (0.067 mol/L, pH  6.8) to prepare 1% starch solution. Next, 50 μL of the tissue homogenate to be tested and 50 μL of the starch solution were mixed and placed in a water bath at 25 ℃ for 3 min. Then, 200 μL of 3,5-dinitrosalicylic acid indicator was added and placed in a boiling water bath for 5 min. The indicator was cooled to 1 mL with running water, and maltose content was determined using colorimetry at 540 nm. Enzyme activity (U/mg prot) was defined as the catalytic hydrolysis of starch to 1 μg maltose by amylase per minute per mg protein.

Protein concentrations (mg/ml) of the enzyme solutions were determined using a protein quantification kit (Coomassie brilliant blue) produced by Jiancheng Institute of Biotechnology (Nanjing, China).

### Statistical analyses

All the data variables were expressed as mean ± standard error (SEM) values. Differences in the investigated variables (water salinity and number of crabs) with respect to different areas, osmolality and enzyme activities (Na^+^/K^+^-ATPase, lysozyme, alkaline phosphatase, phenoloxidase, trypsin, amylase and lipase) were analysed using one-way ANOVA, followed by Tukey’s tests. Independent-samples *t*-test was used to detect differences between the male and female crabs. The relationship between the number of crabs and water salinity was evaluated with linear regression analysis. Statistically significant differences were accepted at *P* < 0.05, and all statistical analyses were performed using the SPSS v19.0 software.
